# Testicular tissue cryopreservation for fertility preservation in prepubertal and adolescent boys: A 6 year experience from a Swiss multi-center network

**DOI:** 10.3389/fped.2022.909000

**Published:** 2022-09-06

**Authors:** Dehlia Moussaoui, Anna Surbone, Cécile Adam, Tamara Diesch-Furlanetto, Céline Girardin, Julie Bénard, Isabelle Vidal, Fanette Bernard, Kanete Busiah, Thérèse Bouthors, Marie-Pierre Primi, Marc Ansari, Nicolas Vulliemoz, Fabienne Gumy-Pause

**Affiliations:** ^1^Division of General Pediatrics, Department of Woman, Child and Adolescent Medicine, Geneva University Hospitals, Geneva, Switzerland; ^2^Fertility Medicine and Gynaecologic Endocrinology Unit, Department Woman-Mother-Child, Lausanne University Hospital, Lausanne, Switzerland; ^3^Oncology and Hematology Unit, Service of Pediatrics, Department Woman-Mother-Child, Lausanne University Hospital, Lausanne, Switzerland; ^4^Division of Pediatric Oncology-Hematology, University Children's Hospital of Basel, Basel, Switzerland; ^5^Pediatric Endocrine and Diabetes Unit, Department of Woman, Child and Adolescent Medicine, Geneva University Hospitals, Geneva, Switzerland; ^6^Unit for Reproductive Medicine and Gynecological Endocrinology, Department of Woman, Child and Adolescent Medicine, Geneva University Hospitals, Geneva, Switzerland; ^7^Division of Pediatric Surgery, Department of Woman, Child and Adolescent Medicine, University Center of Pediatric Surgery of Western Switzerland, Geneva University Hospitals, Geneva, Switzerland; ^8^Pediatric Oncology and Hematology Unit, Department of Women Child and Adolescent, University Hospitals of Geneva, Geneva, Switzerland; ^9^CANSEARCH Research Platform for Pediatric Oncology and Hematology, Department of Pediatrics, Gynecology and Obstetrics, Faculty of Medicine, University of Geneva, Geneva, Switzerland; ^10^Pediatric Endocrinology, Diabetology and Obesity Unit, Service of Pediatrics, Department Woman-Mother-Child, Lausanne University Hospital, Lausanne, Switzerland; ^11^Laboratory of Andrology and Reproductive Biology, Department Woman-Mother-Child, Lausanne University Hospital, Lausanne, Switzerland

**Keywords:** testicular tissue cryopreservation, fertility preservation, prepubertal boys, oncology, gonadotoxicity

## Abstract

Testicular tissue cryopreservation is the only option of fertility preservation in prepubertal boys. While it is considered experimental, since procedures to obtain mature spermatozoa from prepubertal testicular tissue are still under development, testicular tissue cryopreservation programs have emerged worldwide. Our aim was to study the feasibility and safety of a program of testicular tissue cryopreservation in prepubertal and adolescent boys facing gonadotoxic treatment in three University hospitals in Switzerland. Testicular tissue cryopreservation was accepted by 90% of families, with a total of 35 patients included. The average patient age was 8.5 years (range 7 months to 18.5 years). Malignancies were the most common diagnosis (31 patients, 88.6%) with 16 (45.7%) solid tumors and 15 (42.9%) hematological malignancies. Four (11.4%) patients had a benign condition. The main indication for testicular tissue cryopreservation was conditioning for hematologic stem cell transplantation (25 patients, 71.4%). Testicular tissue was cryopreserved according to the freezing protocol of Louvain Catholic University (Belgium), which includes either only immature testicular tissue freezing, or mature and immature testicular tissue freezing depending on the age of the patient and the presence or absence of haploid cells. The median number of spermatogonia per tubule cross-section was 2 (range 0–6) and spermatozoa were found in only one patient. Tumoral cells were found in one testicular biopsy of a leukemic patient. There were two minor adverse events and none of them required medical treatment or surgical revision. Five patients died during follow-up. Our data demonstrate the feasibility and safety of a program of testicular tissue cryopreservation coordinated by a multidisciplinary team of fertility preservation. Despite the experimental aspect of the procedure, the acceptation rate was high, which highlights the willingness of families and patients to participate in testicular tissue cryopreservation.

## Introduction

The development of oncologic treatments has allowed for significant improvement of life expectancy and survival in children diagnosed with cancer (1). The survivors of these oncologic therapies, however, can experience long-term side effects including infertility caused by impaired spermatozoa production including azoospermia (2–4). These well-described long-term effects are related to the gonadotoxic oncologic treatments such as chemotherapy and localized radiotherapy. In prepubertal and adolescent boys, where mature spermatozoa cannot be cryopreserved, the only option for fertility preservation in cases requiring gonadotoxic therapy is testicular tissue cryopreservation (TTC) (5). The goal of TTC is to preserve spermatogonial stem cells. Although this technique is considered experimental as it has not yet been possible to produce mature spermatozoa (with reproductive potential) from human spermatogonia, the recent birth of a female non-human primate following autografting of cryopreserved immature testicular tissue represents a major step to support TTC in prepubertal and adolescent boys (6).

TTC in boys has been discussed for more than 20 years but remains an ethical and legal challenge (7). Fertility preservation programs in prepubertal boys have emerged worldwide but data regarding the outcomes are still limited. In this article, we present 6 years of experience of fertility preservation in prepubertal and adolescent boys in a multi-center network.

## Materials and methods

### Patients and study design

Data were prospectively collected from patients between 0 and 19 years of age who underwent TTC at the Lausanne University Hospital (CHUV), Geneva University Hospitals (HUG), and Basel University Children's Hospital (UKBB) in Switzerland between 2015 and 2020. The indication for TTC was reviewed by a multidisciplinary team dedicated to fertility preservation and the procedure was offered to the family after reaching consensus. Written informed consent was obtained from the guardian(s) prior to inclusion in the study or from the patient if deemed to have consent capacity.

Eligibility criteria are described in Table 1. Chemotherapeutic agents were classified in high and respectively low gonadotoxic drugs, based on the recommendations of the Oncofertility consortium (8), CECOS (Centre d'étude et de conservation des oeufs et du sperme) (9) and on the literature (10–14). Collected data included patient age, pubertal development according to Tanner stages, diagnosis, indication for TTC, and previous exposure to chemotherapy and radiotherapy. The Tanner stage was evaluated by the pediatrician in charge of the child or by the endocrinologist using the pubic hair staging (15). Alkylating chemotherapy exposure was calculated using the cyclophosphamide equivalent dose (CED) calculator (11). The amount of collected testicular tissue, adverse events and living status were reported.

**Table 1:** Inclusion and exclusion criteria for testicular tissue cryopreservation.

**Inclusion criteria**
Prepubertal boys (Tanner 1) greater than 3 months of age
Peri and post-pubertal boys (Tanner 2–4 and Tanner 5 respectively) with unsuccessful or impossible cryopreservation of mature sperm
Scheduled to undergo high-risk gonadotoxic treatment such as:
• High dose alkylating chemotherapy
• High dose cisplatine
• Testicular radiation
• Total body irradiation
Consensus of the multidisciplinary team dedicated to fertility preservation
**Exclusion criteria**
Less than 3 months of age
Guardian or patient refusal
Non high-risk gonadotoxic treatment

Chemotherapeutic agents were classified in high respectively low gonadotoxic drugs, based on the recommendations of the Oncofertility consortium (8), CECOS (Centre d'étude et de conservation des oeufs et du sperme) (9) and on the literature (10–14).

### Tissue retrieval, transportation, and cryopreservation

Testicular tissue was obtained by a unilateral open testicular biopsy performed by a trained, pediatric surgeon under general anesthesia. Whenever possible, the testicular biopsy was performed at the same time as another surgical procedure requiring general anesthesia. Less than one third of the unilateral testicular volume was retrieved. Testicular tissue samples were transferred to Falcon tube (50 ml, Ref. 352098) containing a phosphate-buffered solution (PBS) at 4°C and transported on ice to the Laboratory of Andrology and Reproductive Biology (LABR) of Lausanne University Hospital, which centralized all samples from the three centers. The maximum transport time was 4 hours. There was no temperature monitoring during transport.

For boys younger than 10 years old, testicular tissue was cryopreserved according to the immature testicular tissue freezing protocol of Louvain Catholic University, Belgium (16). On arrival at the laboratory, in no more than 10 min, the biopsy was transferred in a Petri dish positioned on ice (4°C), was divided in 1–2 mm^3^ fragments, which were then placed in cryotubes containing 1 ml of the cryoprotectant solution (Sucrose 0.1 ml/l, DMSO 0.7 mol/l, HSA10 mg/ml). Once the cryotubes were sealed (SYMS III, Cryo Bio System, France), the slow freezing was performed using a programmable freezer (FREEZAL, Air Liquide, Carbagas, Suisse). At the end of the freezing program, the cryotubes were stored at −196°C in liquid nitrogen. A fragment of the extracted tissue was fixed in Formaldehyde 10% and sent to pathology department for histological examination on Haematoxylin-Eosin stained slides. Additional immunohistochemical staining was performed to assess the presence of spermatogonia using specific markers (SALL4 and CD117) and to detect tumoral cells (antibodies according to the underlying disease). Spermatogonia counting was performed per tubule cross-section. In average, 20 seminiferous tubule cross sections were counted. For boys above the age of 10, the method of cryopreservation was defined after tissue analysis according to the protocol of Louvain Catholic University: if haploid cells were observed, half of the sample was cryopreserved according to the mature testicular tissue freezing protocol (17), and the other half according to the immature testicular tissue freezing protocol to increase the chance of subsequent fertility restoration. The mature testicular tissue freezing protocol consists of the mincing of the tissue in a Petri dish and decantation of the solution with G-MOPS-PLUS for 10 minutes. The cell suspension is placed in a first tube. The supernatant is removed, placed in a second tube and centrifugated at 300 g for 10 min. The supernatant is disposed and the cell suspension from the first and the second tubes are mixed. A droplet of this suspension is aspirated and evaluated for spermatozoa counting. An equal amount of freezing medium (Irvine Scientific, No. 9971) is added to the cell suspension and aliquots of 0.5ml are transferred into high security straws (CryoBioSystem, France) which are then sealed in both ends. The straws are placed in a programmable freezer (FREEZAL, Air Liquide, Carbagas, Suisse), which gradually lowers the temperature from 20 to −150°C, and then transferred to cryotanks filled with liquid nitrogen for storage at −196°C. If no mature cells were observed, mature and immature testicular tissue freezing was only completed for boys older than 12 and only immature testicular tissue freezing was done for boys younger than 12.

Apart from the fragment sent to pathology, all other fragments were destined for future clinical use. In case of death, the tissue was either destroyed or conserved in an anonymized fashion for research purposes if the consenting individual(s) had signed the corresponding consent.

### Statistics

Statistical analyses were performed using STATA software (version 16.0). Mean, median and percentages were calculated to describe patient characteristics, indications for fertility preservation, treatment exposure before testicular cryopreservation, and the amount of collected testicular tissue. The Mann-Whitney U test was used for the comparison of spermatogonia count. A two-sided *P*-value of < 0.05 was considered to be significant.

### Study approval/ethics

This study was approved by the local ethics committee (PB_2016-01378) and registered with clinicaltrials.gov (NCT03180918). Each center's protocol was also approved by their respective Institutional Review Board.

## Results

TTC was indicated and offered to 40 patients. Four families declined the procedure. In one case, TTC was accepted by the parents but not completed due to urgency of hematologic stem cell transplantation (HSCT).

Testicular tissue from 35 patients was collected and cryopreserved between April 2015 and September 2020 (Table 2). During the collection period we observed a progressive increase in the number of TTC procedures with the exception of 2020 (3 in 2015, 3 in 2016, 4 in 2017, 8 in 2018, 11 in 2019 and 6 in 2020).

**Table 2:** Patient characteristics, indication for testicular tissue cryopreservation (TTC), treatment received before TTC, amount of retrieved tissue, and clinical complications. CED exposure was based on the cyclophosphamide equivalent dose calculator (11).

	**Patient characteristic**			**Indication to fertility preservation**				**Treatment received before testicular tissue cryopreservation**				**Testicular tissue cryopreservation**			
**Diagnosis**	**Number of patients,** ***n* (%)**	**Age (y),** **mean (SD,** **range)**	**Prepubertal^c^** ***n* (%)**	**Conditioning for** **HSCT, chemotherapy** **alone, *n* (%)**	**Conditioning for** **HSCT, chemotherapy** **and radiotherapy,** ***n* (%)**	**High dose** **chemotherapy,** ***n* (%)**	**Local** **radiotherapy,** ***n* (%)**	**Expected** **CED exposure** **(mg/m^**2**^),** **mean (SD, range)**	**Previous exposure** **to chemotherapy,** ***n* (%)**	**Previous exposure** **to alkylating** **chemotherapy,** ***n* (%)**	**Previous exposure** **to radiotherapy,** ***n* (%)**	**Previous CED** **exposure** **(mg/m^**2**^),** **mean (SD,** **range)**	**Number of** **testicular tissue** **fragments,** **median (SD)**	**Volume of** **testicular tissue** **biopsy (mm^**3**^)** **, median** **(range)**	**Number of** **spermatogonia/** **cross section,** **median** **(range)**	**Clinical** **complications**
**Malignancies**	31 (88.6)	8.6 (5.3, 0.5–18.5)	21 (67.7)	14 (45.2)	7 (22.6)	9 (29)	1 (3.2)	13,002 (11,756, 100–61,200)	19 (61.3)	16 (51.6)	2 (6.5)^b^	5,466 (3,362, 2,000–15,576)	27 (12–60)	54 (24–120)	2 (0–6)	1 minor hematoma
*Hematological malignancies*	15 (42.9)	10.4 (5.6, 0.7–18.5)	8 (53.3)	8 (53.3)	7 (46.7)	0	0	8,365 (5,403, 100–18,388)	13 (86.7)	11 (73.3)	0 (0)	4,342 (1,394, 2,000–7,400)	39 (16–60)	78 (32–120)	1 (0–6)	1 minor hematoma
*Solid Tumors*	16 (45.7)	6.8 (4.5, 0.5–14.7)	13 (81.3)	6 (37.5)	0	9 (56.3)	1 (6.3)	16,212 (14,322, 8,892–61,200)	6 (37.5)	5 (31.3)	2 (12.5)^b^	7,940 (4,906, 3,125–15,576)	26 (12–55)	52 (24–110)	2.5 (0–5)	0
**Benign conditions^a^**	4 (11.4)	7.9 (2.9, 5.5–12)	3 (75)	4 (100)	0	0	0 (0)	1,1012 (5,446, 9,388–12,000)	0 (0)	0 (0)	0 (0)	0 (0)	31 (19–40)	62 (38–80)	2.5 (1–3.5)	1 minor wound dehiscence
Total	35 (100)	8.5 (5.1, 0.5–18.5)	24 (68.6)	18 (51.4)	7 (20)	9 (25.7)	1 (2.9)	12,696 (11,059, 100–61,200)	19 (54.3)	16 (45.7)	2 (5.7)	5,466 (3,362, 2,000–15,576)	29 (12–60)	57 (24–120)	2 (0–6)	2

a Sickle cell disease and thalassemia.

b Cranio-spinal irradiation (medulloblastoma).

c Tanner 1.

HSCT, hematopoietic stem cell transplantation.

CED, Cyclophosphamide equivalent dose.

**Figure 1:**
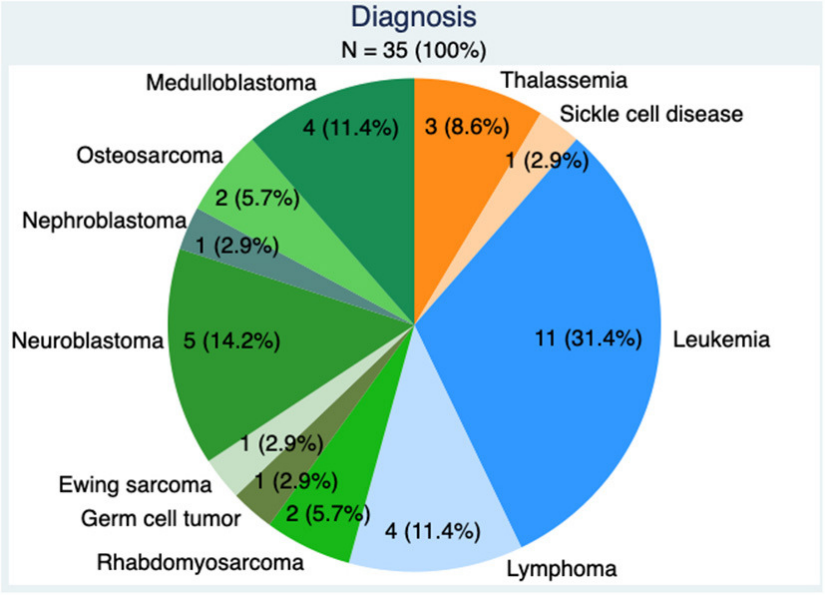
Diagnosis in the 35 patients, benign conditions in orange, hematological malignancies in blue, and solid tumors in green.

The mean patient age was 8.5 years (SD 5.1) and ranged from 7 months to 18.5 years. Twenty-four (69%) patients were prepubertal (Tanner 1), while 9 (26%) were on ongoing puberty (Tanner 2-4). Two boys (5%) with completed puberty (Tanner 5) underwent TTC due to the inability to provide a semen sample by masturbation. Underlying diagnoses requiring gonadotoxic therapy were a malignant disorder in 31 patients including 15 (42.9%) hematological malignancies and 16 (45.7%) solid tumors (Figure 1). Four (11.4%) patients had a benign condition. The primary indication for TTC was conditioning for HSCT (25 patients, 71.4%). Among patients with solid tumors, 7 underwent TTC because of gonadotoxic chemotherapy and radiation (3 medulloblastoma, 1 germ cell tumor, 2 rhabdomyosarcoma, and 1 Ewing sarcoma), 2 because of high dose cisplatine (2 osteosarcoma), and one because of testicular radiation (nephroblastoma stage IV). Nineteen patients (54.3%) had already been exposed to chemotherapy before testicular biopsy, including 16 (45.7%) to alkylating chemotherapy. Average previous CED exposure was 5,466 mg/m^2^ (SD 3,362, range 2,000–15,576 mg/m^2^).

In 23 patients (65.7%), the testicular biopsy was performed at the same time as another surgical procedure requiring general anesthesia. Adverse events were rare: one patient suffered from a minor hematoma and another from a minor wound dehiscence. None of them required medical treatment or surgical revision. The number of testicular tissue fragments varied throughout our series with a median of 29 fragments (range 12–60), corresponding to a median volume of the testicular biopsy of 57 mm^3^ (range 24–120 mm^3^). The median number of spermatogonia per tubule cross-section was 2 (range 0–6). In patients having received alkylating chemotherapy prior to TTC, the median number of spermatogonia was significantly lower than in patients who had not yet received alkylating chemotherapy (0.5 with a range 0–4, and 2.75 with a range 0–6 respectively, *p* = 0.0017). Spermatozoa were found in one patient, aged 15 and who had not received any prior chemotherapy. Based on histology and immunohistochemistry, tumoral cells were found in one testicular biopsy of a leukemic infant. This patient was diagnosed with B-ALL MLL+ at the age of 5 months and treated according to INTERFANT-06 protocol. He was in complete remission with negative bone marrow minimal residual disease (BM-MRD) at the end of induction. However, at the start of MARMA phase, cerebrospinal fluid showed blasts and central nervous system treatment was reinforced before HSCT. BM-MRD was negative before HSCT. The results of the testicular biopsy came after the HSCT and showed leukemic infiltration although he had no clinical sign of testicular involvement. Bilateral testicular biopsies were performed 1 month after HSCT showing no leukemic cells at the immunohistochemical evaluation.

During follow-up five patients died due to tumor progression. Testicular tissue was destroyed in two cases and preserved in three cases, according to the preferences indicated at the time of consent.

## Discussion

This prospective study describes 6 years of experience with pre-pubertal and pubertal TTC. Data from 35 patients was reported, which represents a large prospective series on pre-pubertal and pubertal TTC.

Testicular biopsies were performed in 3 Swiss university hospitals after review by a multidisciplinary team dedicated to fertility preservation. The multicenter design allows for generalization of the findings as well as operator-dependent outcomes (such as complication rate or sample quality) are limited. Similar to other studies, pediatric surgeons removed <1/3 of the entire testicular volume (18, 19).

A single laboratory performed all freezing procedures using a well-validated protocol thus limiting variability in sample handling (20). The centralization of all cryopreservation procedures in a single laboratory could, however, raise concerns regarding sample stability and the optimal timing between surgery and biopsy cryopreservation. As all samples were immediately stored at 4°C in a phosphate-buffered medium, the time elapsed between surgery and tissue manipulation at LABR should have not affected the biopsy quality, as demonstrated by Faes and Goossens in 2016 (21). In their study, testicular tissue could be preserved up to 3 days at 4°C without altering the characteristics of gonadal and somatic cells.

Currently, in prepubertal boys, the only option for fertility preservation is immature testicular tissue biopsy. The present study includes malignant (22) and benign (4) conditions, all requiring a highly gonadotoxic treatment. Interestingly, during the study period, we observed an exponential increase in the number of TTC procedures, with the exception of 2020. This demonstrates successful implementation of an efficient fertility preservation program in boys at the university hospitals involved in the study. The reduction of cases in 2020 might be related to the cancellation of non-urgent HSCT in the context of the COVID-19 pandemic or to fluctuations over time.

Fertility preservation by human immature testicular tissue biopsy is, at present, still experimental as immature testicular tissue was not used *in vitro* or *in vivo* (after grafting) to produce mature spermatozoa with a real reproductive potential.

The easiest option for immature testicular tissue use is by autografting. In patients with malignant disease, examination by molecular techniques of the tissue is mandatory to exclude reintroduction of malignant cells, whereas in non-malignant diseases like hemoglobinopathies there is no restriction for transplantation of the tissue. However, methods for detection of minimal residual disease as multicolor flow cytometry, RT-qPCR, next-generation sequencing and xenograft of tissue in immunodeficient mice are still in development in this context, mainly in ovarian tissue preservation, and validated strategies to ensure the complete safety are still lacking (23–25). A birth *via* autografting has been achieved in non-human primates, indicating the potential feasibility of this technique in humans (6). *In vitro* production of spermatozoa would nevertheless represent the ideal approach as it would avoid the potential risk of reimplantation of tumoral cells associated with autografting. *In vitro* production of spermatozoa is the focus of intense research with several steps successfully realized in the recent years (26). A study published in 2018 demonstrated the feasibility of generating haploid germ cells from immature testicular tissue in organotypic cells cultures, but the real reproductive potential of these cells is still to be demonstrated (27). Another option to avoid the risk of cancer cells contamination is the *in vivo* development of testicular organoids, which allows for prior cell selection. Different studies have recently reported the successful development of testicular function units in rats and pigs (28–31). Research is still ongoing to evaluate the ideal culture medium and conditions necessary to obtain sustained testicular architecture and function (22).

All but four families consented to testicular tissue biopsy, which represents an acceptance rate of 90%, in alignment with what has been reported in the literature (32–34). The decline reason for the four patients was not documented, but the experimental aspect of the procedure may have played a role. To increase parental acceptance and because the procedure is still experimental, the testicular biopsy was coordinated, when possible, with another surgical procedure, to avoid additional exposure to anesthesia and surgical risks for the sole purpose of fertility preservation.

In this series, only a few minor complications occurred (2/35 = 5.7%), demonstrating the safety of this procedure. This finding aligns with previous reports, including larger series (Kanbar et al. reported a complication rate of 3/139 = 2.1%) (20).

One theoretical concern is the presence of tumoral cells within the testicular tissue. This is of special concern in malignancies with a high rate of cancer cell dissemination, for example in leukemia, which represents the majority of the malignancies in our series. A retrospective study found a rate of malignant cells contamination in the testis of boys affected by acute lymphoblastic leukemia as high as 30% (35). Despite this, in the present study, tumoral cell contamination of testicular tissue was found on histology and immunohistochemistry in only one case (a case of acute lymphoblastic leukemia with MLL rearrangement), even though the patient had already been treated by chemotherapy prior to the fertility preservation procedure. Nevertheless, the detection sensitivity of tumoral cells in testicular tissue could be certainly improved by using molecular techniques similar to those used for the quantification of the minimal residual disease.

In our series, 61.3% of the patients with a malignancy had already been treated by chemotherapy at the time of testicular tissue cryopreservation, with 51.6% having received an alkylating chemotherapy at an average CED (cyclophosphamide equivalent dose) of 5,466 mg/m^2^ (range 2,000–15,576). This finding contrasts with the results presented by Kanbar et al., where only 7% of the patients had already received a gonadotoxic therapy prior to the fertility preservation procedure (20). On the contrary, in 2019 Valli-Pulaski et al. described the results of their eight-year experience with pre-pubertal boys fertility preservation programs in several recruitment centers in USA and abroad (19). In their series, 39% of the children had already received a gonadotoxic treatment at the time of testicular tissue biopsy, although at a lower mean dose than in our study (average CED = 2,821 mg/m^2^, range 500–7,000).

Although only based on histology and immunohistochemistry and not on molecular biology, the low rate of tumoral cell contamination of testicular tissue in our series may be due to the high proportion of patients having received a prior chemotherapy, as suggested by Borgström et al. in a recent paper (18). In their series of 21 prepubertal boys undergoing TTC prior to HSCT, including 20 patients with a malignant disease, of which 10 with a leukemia, histopathological analysis found leukemia cells in only one patient. In their opinion, the best time for testicular biopsy in acute lymphocytic leukemia is just before HSCT, when circulating blasts have already been eliminated by the previous chemotherapy. Notwithstanding this, in our series, the only patient with tumoral cell contamination of the testicular sample had previously received chemotherapy.

The real impact of previous chemotherapy on immature testicular tissue sampling, and, in particular, on the quality and number of spermatogonia, is still undefined, even though CED above 4,000 mg/m^2^ could potentially impact spermatogenesis (10). Our study showed a statistically significant difference in the number of spermatogonia according to the prior exposure to alkylating chemotherapy. In the study published by Stukenborg et al., the spermatogonia number per transverse tubular cross-section was significantly reduced in boys exposed to chemotherapy by alkylating agents or hydroxyurea prior to TTC (36). Moreover, Medrano et al. reported a dose-dependent reduction in spermatogonia cells after exposure to alkylating agents, but also cytarabine and asparaginase (37). On the other hand, one study reported no significant difference in spermatogonia number between children previously exposed to gonadotoxic treatment and those who did not receive any previous therapy were observed in testicular samples using immunohistochemistry techniques (19). Recently, normal histology and presence of spermatogonia were observed in testicular tissue even after gonadotoxic therapy and just before conditioning for HSCT in patients, most of them diagnosed with high-risk or relapsed acute lymphoblastic leukemia (18). These results strengthen the concept that an opportunity for fertility preservation should also be offered to children with malignancy relapse or poor response to therapy. In our series the indication for fertility preservation was, in most of the cases, a disease relapse.

The reproductive safety of testicular tissue already exposed to chemotherapy needs to be addressed. Previous exposure to chemotherapy has not been shown to increase the risk of congenital birth defects in offspring of women after ovarian tissue auto-transplantation (38). Children born from childhood cancer survivors have not been found to have an increased rate of chromosomal abnormalities (39). Since no spermatozoa with reproductive potential have been developed from spermatogonial stem cells in humans, the reproductive safety of immature testicular tissue samples exposed to chemotherapy is lacking. Even if data on childhood cancer survivors are reassuring, more studies are needed to assess whether the use of immature testicular tissue cryopreserved after beginning chemotherapy is associated with an increased risk of congenital malformations and adverse neonatal outcomes.

The main limitation of our study is the short duration of follow up, which prevents us from drawing any conclusion on pubertal development and reproductive function after chemotherapy and TTC. Kanbar et al. have reported 139 testicular biopsies performed for fertility preservation between 2005 and 2020, including post-treatment FSH level for 57 patients and post-treatment semen analysis results for 27 of them (20). In those subgroups of patients, they observed higher than normal FSH level in 33% of the 57 patients and severely impaired semen parameters in 52% of the 27 patients. Pubertal onset (defined as a Tanner stage >1 and assessed at the time of the decision to perform the TTC) was an independent factor for testicular insufficiency. The same group has also reported that around 27% of children that complete testicular biopsy will be azoospermic after pubertal transition (26). It is unknown whether the reproductive impairment is merely due to the gonadotoxic therapy or, in part, also to the testicular tissue biopsy itself. In 112 males (median age of 8.6 years) who underwent orchiopexy and bilateral testicular biopsy for unilateral or bilateral undescended testis, reassuring data have shown that the biopsy was not associated with an increased risk of testicular microlithiasis, albuginea scars or testis masse and that no patient had developed antisperm antibody (mean age of 19.6 years) (40). Studies on the reproductive outcomes of children and adolescents treated with gonadotoxic therapies who did not undergo testicular tissue preservation can also help answer this question. In an unselected male population of long-term childhood cancer survivors (after high and low risk gonadotoxic chemotherapy), a high prevalence of oligospermia (20.6%) and azoospermia (17.7%) was observed with a higher prevalence in the high-risk subgroup (41). In a cohort of 55 boys having undergone unilateral testicular biopsy for fertility preservation, testicular volume and growth were similar compared to the contralateral testis at 1, 6 and 12 months of follow-up (42). Despite these reassuring data, more studies and longer follow-up are needed to better clarify issues such as reproductive safety of previously chemotherapy exposed immature testicular tissue and impact of testicular biopsy on pubertal development and reproductive outcomes.

## Conclusion

The present data demonstrates that TTC in prepubertal and adolescent boys represents a safe procedure, with a low immediate complication rate, including in patients with disease relapse and poor response. In addition, the procedure was well-accepted among patients and families, with an acceptation rate of 90%. Although this procedure is experimental and future utilization currently remains hypothetical, TTC represents the only option to preserve future fertility in the pre-pubertal male population and therefore should be offered to patients undergoing highly gonadotoxic treatment.

## Data availability statement

The original contributions presented in the study are included in the article/supplementary material, further inquiries can be directed to the corresponding authors.

## Ethics statement

This study was approved by the Local Ethics Committee (Commission Cantonale d'Ethique de Recherche sur l'être humain—CCER) (PB_2016-01378). Each center's protocol was also approved by their respective Institutional Review Board. Written informed consent to participate in this study was provided by the participants' legal guardian/next of kin.

## Author contributions

CA, TD-F, NV, and FG-P conceived and designed the study and coordinated and supervised the data collection. DM, NV, and FG-P carried out the initial analyses. DM, AS, NV, and FG-P drafted the initial manuscript, reviewed, and revised the manuscript. CA, TD-F, CG, JB, IV, FB, KB, TB, M-PP, and MA critically reviewed the manuscript for important intellectual content and reviewed and revised the manuscript. All authors approved the final manuscript as submitted and agree to be accountable for all aspects of the work.

## Conflict of interest

The authors declare that the research was conducted in the absence of any commercial or financial relationships that could be construed as a potential conflict of interest.

## Publisher's note

All claims expressed in this article are solely those of the authors and do not necessarily represent those of their affiliated organizations, or those of the publisher, the editors and the reviewers. Any product that may be evaluated in this article, or claim that may be made by its manufacturer, is not guaranteed or endorsed by the publisher.
