# Erratum: Testicular tissue cryopreservation for fertility preservation in prepubertal and adolescent boys: A 6 year experience from a Swiss multi-center network

**DOI:** 10.3389/fped.2023.1177389

**Published:** 2023-03-20

**Authors:** Frontiers Production Office

**Affiliations:** Frontiers Media SA, Lausanne, Switzerland

**Keywords:** testicular tissue cryopreservation, fertility preservation, prepubertal boys, oncology, gonadotoxicity

An Erratum on Testicular tissue cryopreservation for fertility preservation in prepubertal and adolescent boys: A 6 year experience from a Swiss multi-center network By Moussaoui D, Surbone A, Adam C, Diesch-Furlanetto T, Girardin C, Bénard J, Vidal I, Bernard F, Busiah K, Bouthors T, Primi M-P, Ansari M, Vulliemoz N and Gumy-Pause F. (2022) Front. Pediatr. 10:909000. doi: 10.3389/fped.2022.909000

An omission to the funding section of the original article was made in error. The following sentence has been added: “Open access funding was provided by the University of Geneva”.

The original version of this article has been updated.

